# Comparison of Minimally Invasive Percutaneous Plate Osteosynthesis and Open Reduction Internal Fixation on Proximal Humeral Fracture in Elder Patients: A Systematic Review and Meta-Analysis

**DOI:** 10.1155/2017/3431609

**Published:** 2017-06-15

**Authors:** Wei Zhao, Yuhui Zhang, Dongni Johansson, Xingyu Chen, Fang Zheng, Liangman Li

**Affiliations:** ^1^Department of Orthopaedics, No. 1 Hospital of China Medical University, Shenyang 110001, China; ^2^Department of Dermatology, No. 1 Hospital of China Medical University, Shenyang 110001, China

## Abstract

**Objective:**

The study aims to compare minimally invasive percutaneous plate osteosynthesis (MIPO) and open reduction internal fixation (ORIF) in the treatment of proximal humeral fracture in elder patients.

**Method:**

PubMed, Medline, EMbase, Ovid, Cochrane Library, China National Knowledge Infrastructure (CNKI), Wangfang, and VIP Database for Chinese Technical Periodicals were searched to identify all relevant studies from inception to October 2016. Data were analyzed with Cochrane Collaboration's Review Manage 5.2.

**Results:**

A total of 630 patients from 8 publications were included in the systematic review and meta-analysis. The pooled results showed that MIPO was superior to ORIF in the treatment of proximal humeral fracture in elder patients. It was reflected in reducing blood loss, operation time, postoperative pain, or fracture healing time of the surgery and in improving recovery of muscle strength. Concerning complications, no significant difference was seen between MIPO and ORIF.

**Conclusion:**

The MIPO was more suitable than ORIF for treating proximal humeral fracture in elder patients.

## 1. Introduction

Proximal humeral fracture is one of the most frequent osteoporotic fractures in the elderly people. The incidence of proximal humeral fracture is increasing with population ages and traffic accidents in urban areas gradually. And women are affected more frequently than men [1].

Therapeutic regimen of proximal humeral fracture should be formulated according to the Neer classification of proximal humeral fracture, which is based on 4 anatomical segments of the proximal humerus and whether these segments are displaced or not [1, 2]. Nondisplaced fractures are commonly treated by conservative treatment; in contrast, displaced fractures are often treated by operations including open reduction internal fixation (ORIF), intramedullary device fixation, minimally invasive plate osteosynthesis (MIPO), or hemiarthroplasty [3].

Of all options, ORIF is a very commonly used method for proximal humeral fracture. Nevertheless, previous studies have shown that there were a raising number of complications with ORIF, including reposition failure, malunion, infection, internal loosening, prosthesis implantation failure, and humerus head vascular necrosis [4]. Recently, minimally invasive plate osteosynthesis (MIPO) has become increasingly popular in the management of fractures [5]. Early clinical studies indicated that MIPO could limit soft tissue injury, reduce operation time, and relieve destruction in blood supply. However, the optimal surgical approach for proximal fracture remains controversial. This systematic review aims to compare MIPO and ORIF for proximal humeral fracture in the elder to provide clinical guidance for surgeons.

## 2. Materials and Methods

### 2.1. Search Strategy

PubMed, Medline, EMbase, Ovid, Cochrane Library, China National Knowledge Infrastructure (CNKI), Wangfang, and VIP Database for Chinese Technical Periodicals were searched to identify relevant studies that compared MIPO with ORIF for the treatment of proximal humeral fractures from inception to October 2016. The search strategy combined the following terms: “proximal humeral/humerus fracture”, “internal fixation/ORIF”, “minimally invasive/MIPO”. Languages and types of articles were not restricted. A manual search was supplemented by verifying the references listed in the key publications.

### 2.2. Study Selection

An overall literature search was performed and relevant studies were screened independently by two reviewers (Wei Zhao, Dongni Johansson). Qualified studies were selected based on the following criteria: ① design of study: randomized controlled study (RCT) or nRCT; ② age of patients: ≥45 years old with proximal humeral fracture; ③ intervention: MIPO and ORIF; ④ at least one of following data having been recorded in articles: intraoperative blood loss, operation time, postoperation pain, complications, fracture healing time, and functional outcomes.

Exclusion criteria are as follows: ① animal model experiment; ② the cases of patient being lower than 10; ③ having no relevant data that could be extracted from articles; ④ duplicate publication.

### 2.3. Data Extraction and Quality Assessment

Relevant data were extracted independently by two reviewers (Wei Zhao, Dongni Johansson). The designed form included the following information: first author's name, publication year, number of patients, mean age, follow-up time, operative type, intraoperative blood loss, operation time, postoperation pain, complications, time of fracture healing, and functional outcomes.

The quality assessment of included studies was independently performed and crosschecked by two reviewers. Disagreements were resolved by discussing with a senior researcher (Liangman Li).

### 2.4. Statistical Analysis

The meta-analysis was conducted using Review Manage 5.2 provided by Cochrane Collaboration. Inverse-variance test was applied for continuous variables and the Mantel-Haenszel test was applied to examine dichotomous variables. The weighted mean difference (WMD) was used to calculate continuous variables across studies that were measured in the same scale. Different scales of continuous variables were combined and calculated by standard mean differences (SMD). Dichotomous variables were carried out by using odds ratio (OR). All data were reported with WMD, SMD, or OR and associated 95% confidence intervals (CI). The heterogeneity between studies was tested by both Chi-square test and *I*-squared test (*I*^2^). *P* < 0.1 or *I*^2^ > 50% was considered as a high heterogeneity between studies. If significant heterogeneity was present (*P* < 0.1, *I*^2^ > 50%), a random-effect model was selected. A fixed-effect model was performed when the significant heterogeneity was absent across studies.

## 3. Results

### 3.1. Literature Search and Study Characteristics

**Figure 1:**
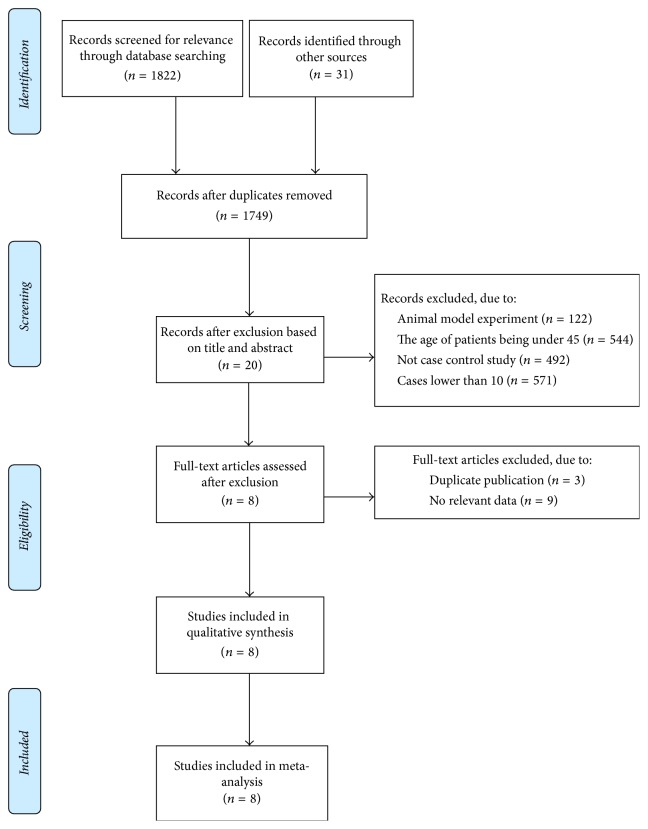
Flow diagram demonstrating those studies which were processed for inclusion.

A total of 1853 relevant studies were retrieved. After removal of duplicates by titles, 1749 articles were further screened. After careful identification, 20 studies were assessed by full-text. Finally, 8 articles met inclusion criteria and were included in the systematic review [3, 4, 6–11]. Selection progress of studies was shown in Figure 1. A total of 630 patients with proximal humeral fracture were involved, including 304 patients treated by MIPO and 326 patients treated by ORIF. Characteristics of patients were listed in Table 1.

### 3.2. Quality Assessment of Included Studies

As all the included studies were nRCT, the quality assessment was conducted by methodological index for nonrandomized studies (MINORS). Methodologic items were as follows: (1) a clearly stated aim; (2) inclusion of consecutive patients; (3) prospective collection of data; (4) end points appropriate to the aim of the study; (5) unbiased assessment of the study endpoint; (6) follow-up period appropriate to the aim of the study; (7) loss to follow-up, which is less than 5%; (8) prospective calculation of the study size; (9) an adequate control group; (10) contemporary groups; (11) baseline equivalence of groups; (12) adequate statistical analyses. The items were scored as “0” (not reported), “1” (reported but inadequate), or “2” (reported and adequate). The global ideal score for comparative studies was 24 [12]. The score over 12 was regarded as high quality. The quality of included studies was presented in Table 2.

### 3.3. Comparison of MIPO and ORIF on Proximal Humeral Fracture

#### 3.3.1. Intraoperative Blood Loss and Operation Time

**Figure 2:**
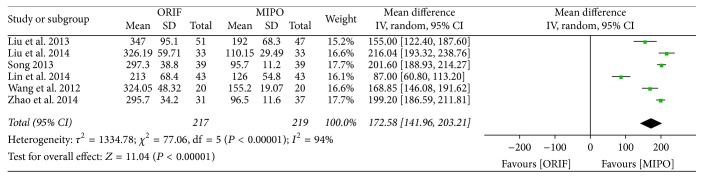
Forest plot of weighted mean difference (WMD) and 95% confidence intervals (CI) for intraoperative blood loss.

**Figure 3:**
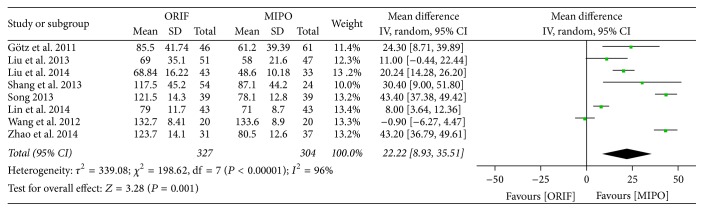
Forest plot of weighted mean difference (WMD) and 95% confidence intervals (CI) for operation time.

Of all the 8 studies, 6 studies which involved 445 cases and all the 8 studies which involved 630 cases, respectively, provided the data on intraoperative blood loss and operation time. There was a significant heterogeneity across studies (intraoperative blood loss: *P* < 0.00001, *I*^2^ = 94%; operation time: *P* < 0.00001, *I*^2^ = 96%), and thus the random-effect models were applied. The meta-analysis indicated that MIPO was superior to ORIF in whatever intraoperative blood loss (WMD = 172.58; 95% CI, 141.96 to 203.21) or operation time (WMD = 22.22; 95% CI, 8.93 to 35.51) (Figures 2 and 3).

#### 3.3.2. Postoperative Pain

**Figure 4:**
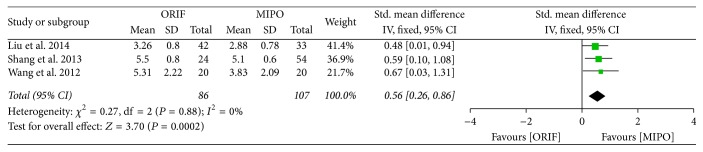
Forest plot of standard mean difference (SMD) and 95% confidence intervals (CI) for postoperative pain of visual analog scale score.

**Figure 5:**
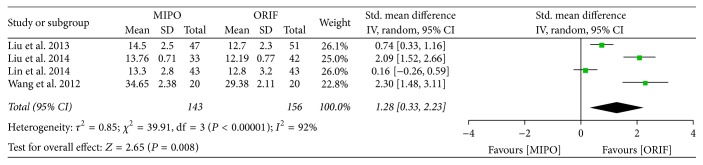
Forest plot of standard mean difference (SMD) and 95% confidence intervals (CI) for postoperative pain of Constant-Murley score.

Visual analog scale (VAS) was used to evaluate short-term postoperative pain in 1 week after surgery. And Constant-Murley score was used to evaluate long-term postoperative pain 6 to 12 months after surgery. Of all included studies, 3 studies provided data on VAS and 4 studies used Constant-Murley score. Statistical analysis of VAS score showed a high homogeneity across studies (*P* = 0.88, *I*^2^ = 0%), and thus the fixed-effect model was used for meta-analysis (Figure 4). However, the Constant-Murley score showed a significant heterogeneity across studies (*P* < 0.00001, *I*^2^ = 92%), and therefore the random-effect model was performed. The meta-analysis in both VAS score (SMD = 0.56; 95% CI, 0.26 to 0.86) and Constant-Murley score (SMD = 1.28; 95% CI, 0.33 to 2.23) showed a better outcome treated by MIPO than by ORIF after surgery. Comparing to ORIF, MIPO effectively reduced postoperative pain (Figures 4 and 5).

#### 3.3.3. Complications

**Figure 6:**
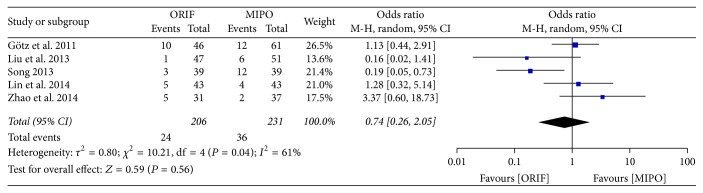
Forest plot of odds ratio (OR) and 95% confidence intervals (CI) for complications.

Complications were available in 5 studies involving 437 patients. A significant heterogeneity was seen across studies (*P* = 0.04, *I*^2^ = 61%), and so the random-effect model was used for meta-analysis. The pooled results showed that no statistical difference was found between ORIF and MIPO treatment (OR = 0.74; 95% CI, 0.26 to 2.05) (Figure 6).

#### 3.3.4. Fracture Healing Time

**Figure 7:**
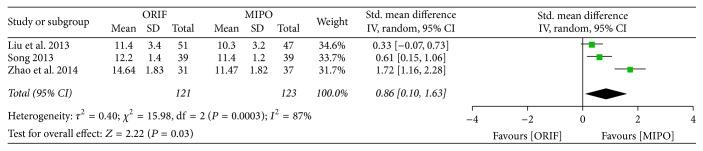
Forest plot of standard mean difference (SMD) and 95% confidence intervals (CI) for fracture healing time.

Fracture healing time was reported in 3 studies, and the result showed that there was a significant heterogeneity among studies (*P* = 0.0003, *I*^2^ = 87%). The meta-analysis conducted by random-effect model indicated that MIPO for proximal humeral fracture had a shorter time of fracture healing compared with ORIF (SMD = 0.86; 95% CI, 0.10 to 1.63) (Figure 7).

#### 3.3.5. Functional Outcomes

**Figure 8:**
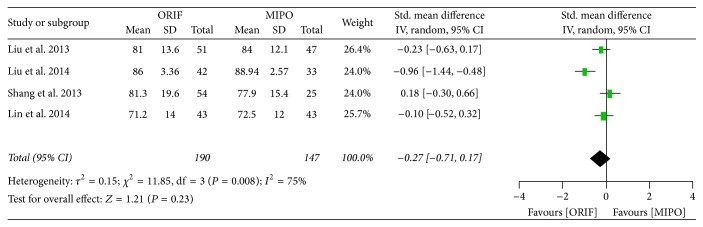
Forest plot of standard mean difference (SMD) and 95% confidence intervals (CI) for functional outcomes of Constant-Murley score.

**Figure 9:**
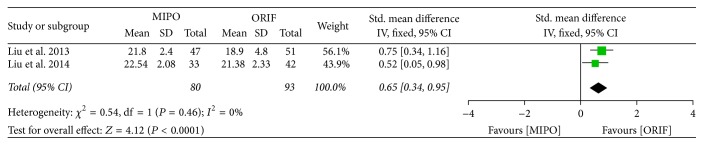
Forest plot of standard mean difference (SMD) and 95% confidence intervals (CI) for muscle strength of Constant-Murley score.

Four articles provided functional outcomes with Constant-Murley score 6 to 12 months after surgery. A significant heterogeneity was seen across studies (*P* = 0.008, *I*^2^ = 75%), and so the random-effect model was used for meta-analysis. The results of overall functional outcomes showed that no significant difference existed between ORIF and MIPO (SMD = −0.27; 95% CI, −0.71 to 0.17) (Figure 8). However, MIPO had a better muscle strength of functional outcome (SMD = 0.65; 95% CI, 0.34 to 0.95) from 6 to 12 months after surgery, indicating that MIPO could improve recovery of muscle strength compared to ORIF (Figure 9).

## 4. Discussion

To our knowledge, this is the first systematic review comparing MIPO and ORIF for the treatment of proximal humerus fracture. The present meta-analysis suggested that MIPO surgical techniques had apparent advantages in intraoperative blood loss, operation time, and fracture healing time compared to ORIF, whereas there was no statistically significant difference between MIPO and ORIF with regard to overall complications. It means that MIPO has minimal risk of operation and is a more appropriate and safer surgical option for elder patients. A shorter operation time and a minimum volume of blood loss could promote postoperative recovery. Furthermore, MIPO treatment for proximal humeral fracture can reduce postoperative pain according to the meta-analysis. An earlier and more effectively functional training would play a positive role for the recovery of motor function. What is more, MIPO had a better outcome of muscle strength scores after operation, enabling patients to engage in early activities and have a better functional exercise. Although these results were obtained, a consensus among orthopedic surgeons on the best treatment for proximal humeral fracture has not been determined [13, 14]. With regard to a range of surgical managements for proximal humeral fracture, the most important goal of the treatment is to restore a normal function without pain at the shortest time. Improvement of functional outcomes and reduction of postoperative pain are the two most considerable aspects in clinical decision.

ORIF technique for proximal humeral fracture is a traditional method by using deltopectoral approach to the proximal humerus. It provides limited access to the posterolateral aspect of the shoulder, and the visualization and reduction of a large retracted greater tuberosity fragment may be difficult [15, 16]. The deltopectoral approach requires extensive soft tissue dissection and muscle retraction to gain adequate exposure to the lateral aspect of humerus. It might increase risks on damage of blood supply to humerus head, and thus the incidence of avascular necrosis could be increased [15, 17]. Gardner and colleagues [17] demonstrated preservation of the humeral head arterial supply with a cadaveric study of MIPO, including the ascending branch of the anterior humeral circumflex vessel and an unnamed posterior branch [5, 18]. Lots of clinical studies have demonstrated the superiority of MIPO to ORIF in preventing the damage of blood supply of the humeral head [19]. Shang et al.'s study [4] suggested that lateral incisions of MIPO provided less soft tissue dissection when the surgery attained reduction of the greater tuberosity. This technique also had an advantage of processing indirect tractive reduction without periosteotomy, which prevented incidence of nonunion and avascular necrosis. MIPO could be a safer surgical option for elder patients due to consideration of biological and physiological conditions of elder patients.

The meta-analysis has made strict inclusion and exclusion criteria, but it still had some limitations and bias which may be unavoidable. No RCTs were involved in this meta-analysis. As a result, subjective factors may affect the result. Different doctors and different hospitals had a variety of surgical technologies and conditions, which may cause bias. The number of included studies and the data for meta-analysis were limited which frustrated the final results to a certain degree. More rigorous designs and large RCTs are required to make further verification.

In conclusion, the pooled results showed that MIPO was superior to ORIF in the treatment of proximal humeral fracture in elder patients. It was reflected in reducing blood loss, operation time, postoperative pain, or fracture healing time of the surgery and in improving recovery of muscle strength. The MIPO was more suitable than ORIF for treating proximal humeral fracture in elder patients.

## Figures and Tables

**Table 1:** Characteristics of the included studies.

Reference	Number of patients	Gender	Mean age	Follow-up	Operative
	MIPO/ORIF	Male/female	MIPO/ORIF	(Months)	Type
Zhao et al.	37/31	26/42	71.3/71.5	12	Neer: 2, 3, 4
Song	39/39	23/55	64.2	12	Neer: 2, 3, 4
Shang et al.	24/54	19/59	61.6/60.0	12	Neer: 2, 3, 4
Wang et al.	20/20	14/26	69.6/69.7	12	Neer: 2, 3
Lin et al.	43/43	28/58	63/61	12	AO: A, B, C
Liu et al.	47/51	43/55	72.8/49.9	12	Neer: 3, 4
Liu et al.	33/42	28/47	47.3/49.1	12	Neer: 2, 3
Götz et al.	61/46	32/75	65/67.6	12	AO: A, B, C

*Note*. Neer's terminology of four-segment classification of displaced fractures and fracture-dislocations relates pattern of displacement (Neer: 2, two-part; Neer: 3, three-part; or Neer: 4, four-part) and key segment displaced. The AO classification is based on the severity of the fracture and the likely disruption to the vascularity of the proximal humerus, including three broad types of fracture. Type A fractures are extra-articular and unifocal, type B fractures are extra-articular and bifocal, and type C fractures are articular.

**Table 2:** MINORS appraisal scores for the included studies.

Study	Methodological items												Total
	1	2	3	4	5	6	7	8	9	10	11	12	
Zhao et al.	2	1	0	1	0	2	2	0	1	2	1	2	14
Song	2	2	0	2	0	2	1	0	2	2	2	2	17
Shang et al.	2	1	0	2	0	2	1	0	2	2	2	2	16
Wang et al.	2	2	0	2	0	2	2	0	2	2	1	2	17
Lin et al.	2	2	0	2	0	2	1	0	2	2	2	2	17
Liu et al.	2	2	0	2	0	2	2	0	1	2	1	2	16
Liu et al.	2	1	0	2	0	2	1	0	2	2	2	2	16
Götz et al.	2	2	0	2	0	2	2	0	2	2	2	2	18
